# Lower spinal postural variability during laptop-work in subjects with cervicogenic headache compared to healthy controls

**DOI:** 10.1038/s41598-021-84457-6

**Published:** 2021-03-04

**Authors:** Sarah Mingels, Wim Dankaerts, Ludo van Etten, Liesbeth Bruckers, Marita Granitzer

**Affiliations:** 1 REVAL Rehabilitation Research Centre, Biomedical Research Institute, Faculty of Rehabilitation Sciences, Hasselt University, 3500 Hasselt, Belgium; 2 Musculoskeletal Research Unit, Department of Rehabilitation Sciences, Faculty of Kinesiology and Rehabilitation Sciences, Leuven University, 3000 Leuven, Belgium; 3 Department of Biometrics, Zuyd Hogeschool, 6419 Heerlen, The Netherlands; 4 Interuniversity Institute for Biostatistics and Statistical Bioinformatics, Hasselt University, 3500 Hasselt, Belgium

**Keywords:** Pain, Spine motility

## Abstract

Spinal postural variability (SPV) is a prerequisite to prevent musculoskeletal complaints during functional tasks. Our objective was to evaluate SPV in cervicogenic headache (CeH) since CeH is characterized by such complaints. A non-randomized repeated-measure design was applied to compare SPV between 18 participants with reporting CeH aged 29–51 years, and 18 matched controls aged 26–52 years during a 30-min-laptop-task. Habitual spinal postures (degrees) of the cervical, thoracic and lumbar spine were analysed using 3D-Vicon motion analysis. SPV, to express variation in mean habitual spinal posture, was deducted from the postural analysis. Mean SPV of each spinal segment was lower in the CeH-group compared to the control-group. Within the CeH-group, SPV of all except one spinal segment (lower-lumbar) was higher compared to the group’s mean SPV. Within the control-group, SPV was more comparable to the group’s mean SPV. SPV differed between groups. Averaging data resulted in decreased SPV in the CeH-group compared to the control-group during the laptop-task. However, the higher within-group-SPV in the CeH-group compared to the group’s mean SPV accentuated more postural heterogeneity. It should be further determined if addressing individual SPV is a relevant intervention.

## Introduction

Motor variability refers to variation in postures, movements and muscle activity needed for adaptation^1, 2^. According to the Neuronal Group Selection Theory, primary and secondary motor variability are characteristics of normal motor development^3^. Primary variability means that during human development the nervous system explores a wide range of motor possibilities, which causes an enormous non-adaptive variation in motor behaviour^4, 5^. Secondary or adaptive variability, i.e. selection of motor behaviour which fits best the situation based on afferent information produced by behaviour and experience, is important to adapt motor behaviour to the task^1, 3, 5^. Such adaptive variability is hypothesized to fulfil a beneficial role in preventing development of overuse injuries and pain^2^. Low motor variability or stereotypical motor behaviour is related to overuse of tissue, which can progress towards peripheral maladaptation such as reduced capillary-to-fibre ratio and mitochondrial disturbance^6^. These processes can facilitate development of musculoskeletal disorders (MSDs) and pain^2, 6^.

The cost of for instance work-related upper-limb MSDs has been estimated to amount between 0.5 and 2% of the country’s Gross National Product^7^. According to the European Agency for Safety and Health at Work (2019), MSDs and exposure to their risk factors (e.g. sedentary behaviour, screen time, psychosocial risk factors) are still increasing^8^.

Within this context, it might be relevant to draw attention to musculoskeletal complaints in cervicogenic headache (CeH). Prevalence of musculoskeletal complaints, such as observed in CeH, increase when daily computer use exceeds 3 h^9, 10^. These complaints are presumed to be related to the rather static habitual slumped sitting posture, characterized by posterior pelvic rotation, thoracic flexion, and especially forward head posture (FHP), during laptop- and desktop-computer use^11, 12^. An increased load on cervical musculoskeletal structures caused by the pronounced FHP might be a possible link with the development and persistence of CeH^13, 14^.

Cervical spine, its structures, and CeH are neuro-anatomically related through the trigemino-cervical complex (TCC)^15^. Its pars caudalis receives first-order nociceptive Aδ and C afferents of the ophthalmic part of the trigeminal nerve together with first-order Aδ and C nociceptive afferents from mostly the C2 dorsal nerve root. Nociceptive inputs from the ophthalmic and C2 nerve root overlap in the TCC. Convergence of trigeminal and cervical Aδ and C fibres on the C1-C2 dorsal horn provides a neuro-anatomical base for referred pain; pain originating from the neck is perceived as headache and vice versa^16, 17^.

Any persisted posture (e.g. FHP) with insufficient variation is a risk factor to develop a painful condition^18, 19^. As a consequence, it might be hypothesized that decreased cervical spinal postural variability (SPV), due to stereotypical motor behaviour and maintaining a posture^2, 7, 20^, might contribute to episodic CeH. However, since the lumbar and cervical spine move jointly in opposite directions during sitting^12^, it seems relevant to look beyond the cervical spine when analysing postural variability. A study by Caneiro et al. (2010) revealed that three defined thoraco-lumbar sitting postures resulted in variations of muscle activation, head and neck kinematics. Slumped sitting for instance was associated with an increased cervical flexion and anterior translation of the head compared to upright sitting^21^. Such slumped thoraco-lumbar posture is assumed to indirectly stress the cervical spine and thereby activating the TCC. Further, based on the contemporary theory of motor adaptation to pain, patients suffering from a painful condition adopt a protective less variable posture, which may even occur in regions remote to the site of pain^22, 23^. Applied to episodic CeH, this implies that the adjacent thoracic and lumbar spine might freeze to protect the cervical spine. Nevertheless, such contra-productive motor behaviour can increase the cumulative load on cervical spinal structures^24^ and thereby activating the TCC.

Therefore, the main objective of this study was to evaluate if SPV differed between patients with episodic CeH and an asymptomatic matched control-group during a laptop-task. The study focussed on higher level aspects (i.e. kinematic models) of human movement behaviour since such analysis provides more realistic and applicable models to analyse complex multi-joint movements^25^.

## Methodology

### Design

Non-randomized longitudinal (repeated-measures) comparison of SPV between the episodic CeH-group and control-group during a 30-min-laptop-task.

### Sample size

Sample size calculation was not performed to establish treatment-effect but rather to assess feasibility of evaluating SPV in the context of an exploratory study. Yet, an a priori sample size was estimated (G*Power 3.1.9.4, Kiel Germany). Based on repeated-measures at five time-points (F-test, between-factors) of the FHP (mean degrees and standard deviation) during a laptop-task, a total of 30 participants (15 participants per group, power 80%; α = 0.05) was required to detect a mean difference of 3.5° (± 1.3) in FHP between the headache-group and control-group^26^.

### Participants

Participants for both the CeH-group and the control-group were recruited between January 2018 to August 2019. The neurological staff at the headache departments of the AZ Vesalius hospitals (Tongeren and Bilzen, Belgium) identified and referred participants meeting the study’s inclusion criteria for CeH (see below for details). Additionally, a general call was launched at the Hasselt University, Zuyd Hogeschool, and private practice of the principal researcher. These potential participants also had to be declared eligible by the neurologist. Potential participants for the control-group were recruited by convenience sampling, word-of-mouth advertising within the Zuyd Hogeschool, and in the personal network of the involved researcher (Appendix a).

*Inclusion criteria for the CeH-group* were: Dutch-speaking participants between 18 and 55 years, body mass index (BMI) between 18.5 and 24.9 kg/m^2^, diagnosed with secondary episodic CeH according to the International Classification of Headache Disorders 3 (ICHD)^27^ by a neurologist, minimum laptop-use of seven hours/week, normal cognitive capacity, headache provocation through manual unilateral posterior-anterior cervical pressure (Passive Accessory Intervertebral Movement) at the C0-C4 region by a manual physiotherapist.

Inclusion criteria for the control-group were: Dutch-speaking asymptomatic participants between 18 and 55 years, BMI between 18.5 and 24.9 kg/m^2^, minimum laptop-use of seven hours/week, normal cognitive capacity.

Exclusion criteria for both groups were: pregnancy, physiotherapy for head- or neck-related disorders in the past month before the start of the study, serious pathology (musculoskeletal, neurological, endocrine, cardiovascular, psychiatric), comorbid headache, pain radiation to the arm(s), medication overuse (intake of non-steroid anti-inflammatory drugs, opioids, acetylsalicylic acid, triptans, simple analgesics for > 10 days/month > 3 months), smoking, history of neck/head trauma, orthodontics.

Nineteen participants were recruited and selected to compose the CeH-group (Appendix a). These participants were given a four-week headache-diary. The control-group was matched for age, gender, ethnicity and socio-economic status (level of education, job).

**Figure 1:**
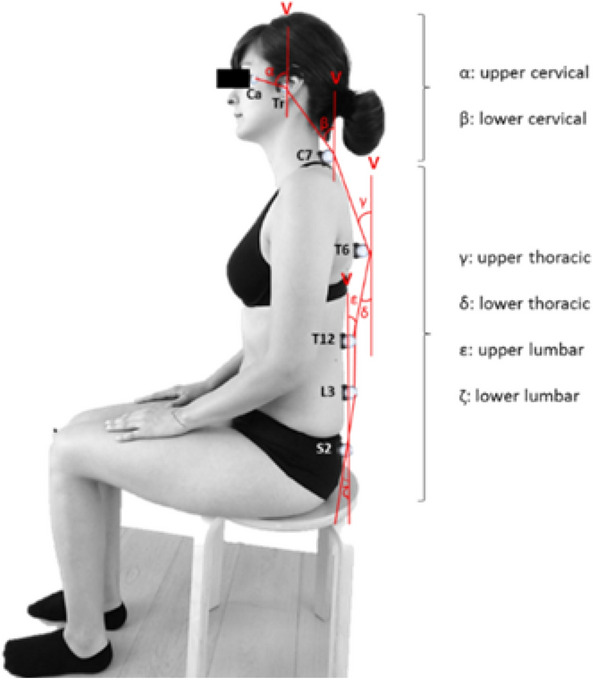
Biomechanical model with marker placement and angle (α to ς) determination (*V* vertical;* Ca* canthus; *Tr* tragus).

The current study is part of phase 1 of a larger project which is registered as an observational study at ClinicalTrials.gov (NCT02887638). The Medisch Ethische ToetsingsCommissie of Zuyderland and Zuyd Hogeschool (NL. 55720.09615) and the Comité Medische Ethiek of the Ziekenhuis Oost-Limburg (B371201423025) granted approval to execute the experimental protocol. Eligible participants had to read and sign the informed consent before officially being enrolled. Protection of personal data is legally determined by the Belgian law of December 8th 1992. All test procedures involving human participants were in accordance with the ethical standards of the institutional research committees and with the 1964 Helsinki Declaration and its later amendments. An informed consent was obtained from the participant in Fig. 1 for publication of the identifying image.

### Measurements, outcomes and instruments

*Primary outcomes.* Habitual spinal postures (= the position of the spine, expressed via degrees, °) of the upper- and lower-cervical (UCx, LCx), thoracic (UTx, LTx), and lumbar (ULx, LLx) spine were evaluated with a 3D-Vicon motion analysis system (Vicon Motion Systems Ltd., Oxford, UK) and Nexus 2.1.1 software (recording, data acquisition, storage, gap filling) during the 30-min-laptop-task^28, 29^. The accuracy of the system is < 1° and < 1.5° root mean square in static and dynamic angular measurements, respectively^30^. SPV of each spinal posture, expressed by the standard deviation (SD), was deducted from the postural measurements of the habitual spinal posture during the 30-min-laptop-task. SPV expresses variation in the mean habitual spinal posture.

#### Secondary outcome

Headache-intensity pre and post laptop-task was measured using the 11-point Numeric Pain Rating Scale (NPRS) which ranges from 0 (no pain) to 10 (worst pain imaginable). Scores ≤ 3 correspond to mild, 4–6 to moderate, and ≥ 7 to severe pain. The meaningful clinically important change amounts to 2.5. Psychometric properties of the NPRS are solid^31^.

### Data collection, processing and analysis

The principal researcher was responsible for the data collection. Motion analysis was performed via 12 infrared Bonita T10 and two video cameras at a sample rate of 100 Hz (low-pass Woltring filter)^32^. The biomechanical model, a custom labelling skeleton template, was created with Vicon Nexus and evaluated (2016–2017, Zuyd Hogeschool). This model was derived from numerous studies that examined human sitting posture^33–39^. Seven reflective markers were placed on the following anatomical landmarks to model the spine: left tragus and canthus, C7, T6, T12, L3 and S2 spinous processes. Data processing was conducted in Vicon Nexus 2.1.1. Processed trials were converted to pseudonymized c3d-files, exported to a custom developed data analysis programme (MATLAB R2019b, Natick, Massachusetts: The MathWorks Inc.), and converted to angles by an independent researcher.

Spinal posture was recorded each minute for five seconds during the 30-min-laptop-task. Angles were calculated based on the mean of the middle three seconds of the five second recording. Left sagittal angles were calculated for the: UCx, LCx, UTx, LTx, ULx, and LLx (Table 1, Fig. 1). SPV was deducted from these measurements.

**Table 1:** Overview of the determination of spinal angles of the biomechanical model.

Angle	Markers (section)	Angle (°)
Upper-cervical (α)	Tragus, canthus (8 mm)	Between line through markers on tragus–canthus and vertical
Lower-cervical (β)	Tragus (8 mm), C7 (14 mm)	Between line through markers on C7—tragus and vertical
Upper-thoracic (γ)	C7, T6 (14 mm)	Between line through markers on T6–C7 and vertical
Lower-thoracic (δ)	T6, T12 (14 mm)	Between line through markers on T12–T6 and vertical
Upper-Lumbar (ε)	T12, L3 (14 mm)	Between line through markers on L3–T12 and vertical
Lower-lumbar (ζ)	L3, S2 (14 mm)	Between line through markers on S2–L3 and vertical

°, degrees; *mm *millimetre.

Spinal angles were calculated based on following method, an example is given for angle $\mathrm{\alpha }$(UCx):

Let Canthus (Ca) be $$\left(\begin{array}{c}{\mathrm{x}}_{\mathrm{ca}}\\ {\mathrm{y}}_{\mathrm{ca}}\\ {\mathrm{z}}_{\mathrm{ca}}\end{array}\right)$$, Tragus (Tr) $$\left(\begin{array}{c}{\mathrm{x}}_{\mathrm{tr}}\\ {\mathrm{y}}_{\mathrm{tr}}\\ {\mathrm{z}}_{\mathrm{tr}}\end{array}\right)$$ and Vertical (V) $$\left(\begin{array}{c}0\\ 0\\ 1\end{array}\right)$$, then vector TrCa is $$\left(\begin{array}{c}{\mathrm{x}}_{\mathrm{tr}}-{\mathrm{x}}_{\mathrm{ca}}\\ {\mathrm{y}}_{\mathrm{tr}}-{\mathrm{y}}_{\mathrm{ca}}\\ {\mathrm{z}}_{\mathrm{tr}}-{\mathrm{z}}_{\mathrm{ca}}\end{array}\right)$$. Now $\mathrm{\alpha }$ can be calculated using Eq. (1).

1 $$\mathrm{cos}\left(\alpha \right)=\frac{TrCa \cdot V}{\Vert TrCa\Vert \Vert V\Vert },$$

with $$\Vert a\Vert =\sqrt{{x}_{a}^{2}+{y}_{a}^{2}+{z}_{a}^{2}}$$.

### Procedure

#### Marker placement and static calibration

Concerning the CeH-group, a condition to be measured was a score of < 3 on the 11-point NPRS for headache-intensity on the test day^22^. Participants were asked not to take analgesics, muscle relaxants, and caffeine-containing beverages 24 h prior to the measurements. Prophylactic treatment(s) remained unchanged. Measurements were performed in a real-life set-up with a constant room temperature of 25° Celsius at the motion laboratory of Zuyd Hogeschool (Heerlen, The Netherlands). A static calibration of the sitting posture was performed for each participant before testing started. During the calibration participants were seated on a desk chair without back support, both feet on the floor, upper legs horizontal, lower legs vertical, feet parallel and shoulder width apart on the ground, arms uncrossed on thighs (Fig. 1)^33, 40, 41^. After degreasing the skin, anatomical landmarks were located by manual palpation, and marked by an experienced (> 10 years) manual therapist (= principal researcher). The most prominent bony anatomical landmarks were selected to limit soft tissue artefacts. Next, reflective markers were fixed on the skin using double-sided adhesive tape at the previously described anatomical landmarks^33–39^. Individual spinal postures were expressed relative to their static sitting posture. The principal researcher performed the test procedure for both the CeH-group and control-group.

#### Workstation setup

The capture volume of the Vicon system was 15 m^2^. A standard desk (height 74 cm; depth 80 cm; width 120 cm) (Bureau voor Normalisatie NBN-EN527) with laptop (HP ProBook 650 or HP EliteBook 6470b) and a height-adjustable office chair without back rest were placed in the centre of the capture volume^42, 43^. The position of the laptop, inclination of the screen and position of the chair referred to the ground could be individually adjusted^44^. The position of the setup had to ensure visibility of each marker by at least two infrared cameras.

#### Test procedure

Participants were asked to *‘sit as you normally do’*. No further instructions, nor feedback was given^45^. Hereafter a 35-min customized laptop-task was performed. Posture was not recorded throughout the first five minutes to familiarize the participant with the work station^46–48^. During the 30-min-laptop-task the habitual spinal posture was recorded for five seconds every minute^34, 49, 50^, the first measurement was defined as ‘t0’. SPV was deducted from these measurements.

For the customized laptop-task the most common on- and offline laptop activities were selected (browsing, editing and typing). Participants completed nine standardised questions (Microsoft Office Word 2016), four questions accentuated typing words and numbers through open questions, five questions required an internet-search (Google Chrome or Mozilla Firefox), the use of a computer-mouse was not allowed^51^. Headache-intensity was questioned (NPRS) pre and post laptop-task.

The test procedure was executed and guided by the principal researcher.

### Statistics

Analysis was done via JMP Pro 14 and SAS 9.4. Two-tailed tests at 5% level of significance were reported.

#### Demographics and group characteristics at baseline

Unpaired t-tests or non-parametric Mann–Whitney Tests, depending on the conditions (normality and equality of variances), were used to compare continuous variables. Contingency tables (Fisher’s exact test) were composed to compared distributions of categorical variables (proportions) between groups.

Mean habitual spinal angles (degrees) at t0 and during the 30-min-laptop-task were presented as means (SD). Linear mixed models for repeated measures were built based on the lowest root-mean-square error, with dependent variables (angles), fixed (time, baseline, group, headache-intensity), random (individual, time) effects, and an autoregressive covariance structure [AR(1)]: Y_ij_ = β_0_ + β_1_X_1ij_ + … + β_k_X_kij_ + μ_i_ + ε_ij_ (Appendix b, Eq. b.1).

(a) Between-group-variability or mean variability at t0 was deducted from the random-intercept of the mixed model and expressed by the SD (= √variance). Mean angles (degrees) and variability of each spinal posture were compared between groups by adding a fixed group effect to the mixed model.

(b) Within-group-variability (i.e. residual variability during the laptop-task) was computed from the squares of the difference between each individual value and the mean value of the group that this individual value has come from. Within-group-variability was additionally used to compare UCx vs. LCx, UTx vs. LTx, and ULx vs. LLx variability.

(c) Intraclass correlation coefficients (ICCs) were calculated to estimate how strongly individuals in the same group (i.e. CeH-group and control-group) resemble each other. ICCs were calculated of the repeated-measurements (30 time-points) of each spinal posture. A 2-way mixed-effects model was composed (fixed effect: time, random effect: individual)^52^. ICCs were interpreted according to Portney et al. (2000): values < 0.50 are indicative of poor, values between 0.50 and 0.75 of moderate, values between 0.75 and 0.90 of good, and values > 0.90 of excellent reliability^53^.

(d) Influence of headache-intensity (NPRS) on SPV was analysed by comparing the random-intercepts (= variance) of each spinal angle between a mixed model with post-laptop-task headache-intensity (independent variable) and a mixed model without post-laptop-task headache-intensity (Appendix d, Table d.2). Only the post-laptop-task headache-intensity was included, since at baseline a condition to participate was NPRS < 3.

**Table 2:** Demographics and group characteristics of the CeH-group (n = 18) and control-group (n = 18).

	CeH-group	Control-group	p
Age (year), mean (SD) [CI]	40.2 (10.9) [34.6;45.8]	39.2 (13.1) [32.7;45.7]	.80^‡^
BMI (kg/m^2^), mean (SD) [CI]	23.5 (3.2) [21.9;25.1]	23.2 (3.2) [21.6;24.8]	.76^‡^
**Marital status, n (%)**			1^†^
Married	9 (50)	9 (50)	
Living together	5 (27.8)	4 (22.2)	
In a relation (not living together)	2 (11.1)	3 (16.7)	
Single	2 (11.1)	2 (11.1)	
**Socioeconomic status, n (%)**			
*Job*			.65^†^
Student	2 (11.1)	3 (16.7)	
Working	16 (88.9)	15 (83.3)	
Services	14 (87.5)	13 (72.2)	
Self-employed	2 (12.5)	2 (12.5)	
*Level of education*			1^†^
Secondary studies	2 (11.1)	2 (11.1)	
Graduate school or university	16 (88.9)	16 (88.9)	
**Dominant hand, n (%)**			.22^†^
Left	3 (16.7)	0	
Right	15 (83.3)	18 (100)	
**Laptop-use, hours a week, n (%)**			.89^†^
Little (< 7 h)	1 (5.6)	1 (5.6)	
Substantiate (7–14 h)	5 (27.8)	6 (33.3)	
Moderate (14–21 h)	1 (5.6)	2 (11.1)	
Severe (> 21 h)	11 (61.1)	9 (50)	
Inclination laptop (°), mean (SD) [CI]	115.3 (5.5) [112.4;118.1]	112.4 (11.3) [106.8;118.1]	.35^‡^
Laptop vs. edge table (cm), mean (SD) [CI]	10.1 (5.5) [7.4;12.8]	9.9 (4.5) [7.7;12.1]	.89^‡^
Sedentary-time work hours a day, mean (SD) [CI]	5 (2.7) [3.7;5.1]	5.5 (2.9) [4.1;7]	.92^Δ^
Sedentary-time free hours a day, mean (SD) [CI]	3.3 (1.7) [2.48;4.19]	3.8 (2.5) [2.6;5.1]	.95 ^Δ^
**Episodic cervicogenic headache, n (%)**	18 (100)	0	N/A
Headache-duration, mean hours/episode (SD) [CI]	4.1 (1.6) [3.3;4.9]	N/A	N/A
Headache-intensity, mean VAS/episode (SD) [CI]	60.9 (14) [54.4;67.4]	N/A	N/A
Headache-frequency, median days/month [IQR]	11 [10;15.8]	N/A	N/A
Neck pain (yes), n (%)	18 (100)	N/A	N/A
NPRS pre-test, mean intensity (SD) [CI]	0.7 (1) [0.3;1.2]*	N/A	N/A
NPRS post-test, mean intensity (SD) [CI]	3.6 (2.1) [2.6;4.7]*	N/A	N/A

*y* years, *n* number participants, *VAS* 100 mm Visual Analogue Scale (0 = no paint, 10 = worst pain), *NPRS*  11-point Numeric Pain Rating Scale, *IQR* 25–75% interquartile range, *N/A*  not applicable.

^‡^Unpaired t-test; ^†^Contingency table for categorical variables (Fisher’s exact test); ^Δ^Mann Whitney Test; *NPRS pre laptop-task was significantly (p < .0001, Wilcoxon Signed Rank test) lower (effect size = 0.89) compared to NPRS post laptop-task. Data on headache characteristics were deducted from the 4-week headache-diary.

Mean (SD) headache-intensity (NPRS) was compared pre and post the laptop-task by using the non-parametric Wilcoxon signed-rank test. Effect sizes (ES) to quantify differences in change of headache-intensity from pre- to post-laptop-task were reported and interpreted as follows: ≤ 0.10 small, 0.21 to 0.29 medium, 0.30 to 0.49 large, ≥ 0.70 very large ES^54^.

Relations between the independent variables age, BMI, their interaction (continuous), socioeconomic status (categorical), and postural variables UCx, LCx, UTx, LTx, ULx, and LLx angles (dependent continuous) were evaluated via multiple linear regression: Y_i_ = β_0_ + β_1_X_i1_ + β_2_X_i2_ + β_3_X_i1_X_i2_ + … + β_k_X_ik_ + ε_i_ (Appendix b, Eq. b.2). Conditions to apply linear models had to be met.

## Results

### Demographics and group characteristics

Demographics and group characteristics were comparable between groups, excluding headache characteristics (Table 2). One patient in the CeH-group (h,9) had to be excluded from the data-set because of technical artefacts. Age, BMI, their interaction, level of education, and employment did not significantly influence habitual spinal posture or SPV (Appendix c).

### Does spinal postural variability differ between patients with episodic CeH and controls (Table 3, Fig. 2, Appendix d)?

**Table 3:** Spinal postural variability and ICC in the CeH-group and control-group.

	Mean SPV, SD [CI]		SPV within the group, SD [CI]		ICC [CI]	
	CeH-group	Control-group	CeH-group	Control-group	CeH-group	Control-group
UCx	7.2 [6.7; 7.8]	11.2 [10.5; 12]	14.1 [13.3; 15.1]	11.1 [10.5; 11.9]	0.30 [− 1.3; 1.9]	0.99 [− 1.7; 3.7]
LCx	6.7 [6.3; 7.3]	13.9 [13; 14.9]	15.2 [14.3; 16.2]	13.7 [12.9; 14.6]	0.24 [− 1.2; 1.7]	0.99 [− 0.5; 2.5]
UTx	3.8 [3.5; 4.1]	4.7 [4.4; 5]	11.4 [10.8; 12.2]	4.6 [4.3; 4.9]	0.77 [0; 1.6]	0.97 [0; 2]
LTx	3.5 [3.2; 3.8]	5.2 [4.9; 5.5]	3.9 [3.7; 4.2]	5.3 [5; 5.6]	0.65 [0.1; 1.2]	0.97 [0.2; 1.8]
ULx	3.3 [3.1; 3.6]	4.1 [3.8; 4.4]	3.4 [3.2; 3.6]	5.2 [4.9; 5.7]	0.73 [0.1; 1.3]	0.81 [0; 1.6]
LLx	9.2 [8.5; 9.9]	11.4 [10.5; 12.5]	8.8 [8.3; 9.4]	10.6 [10; 11.3]	0.70 [− 0.1; 1.5]	0.92 [− 0.8; 2.7]

**Figure 2:**
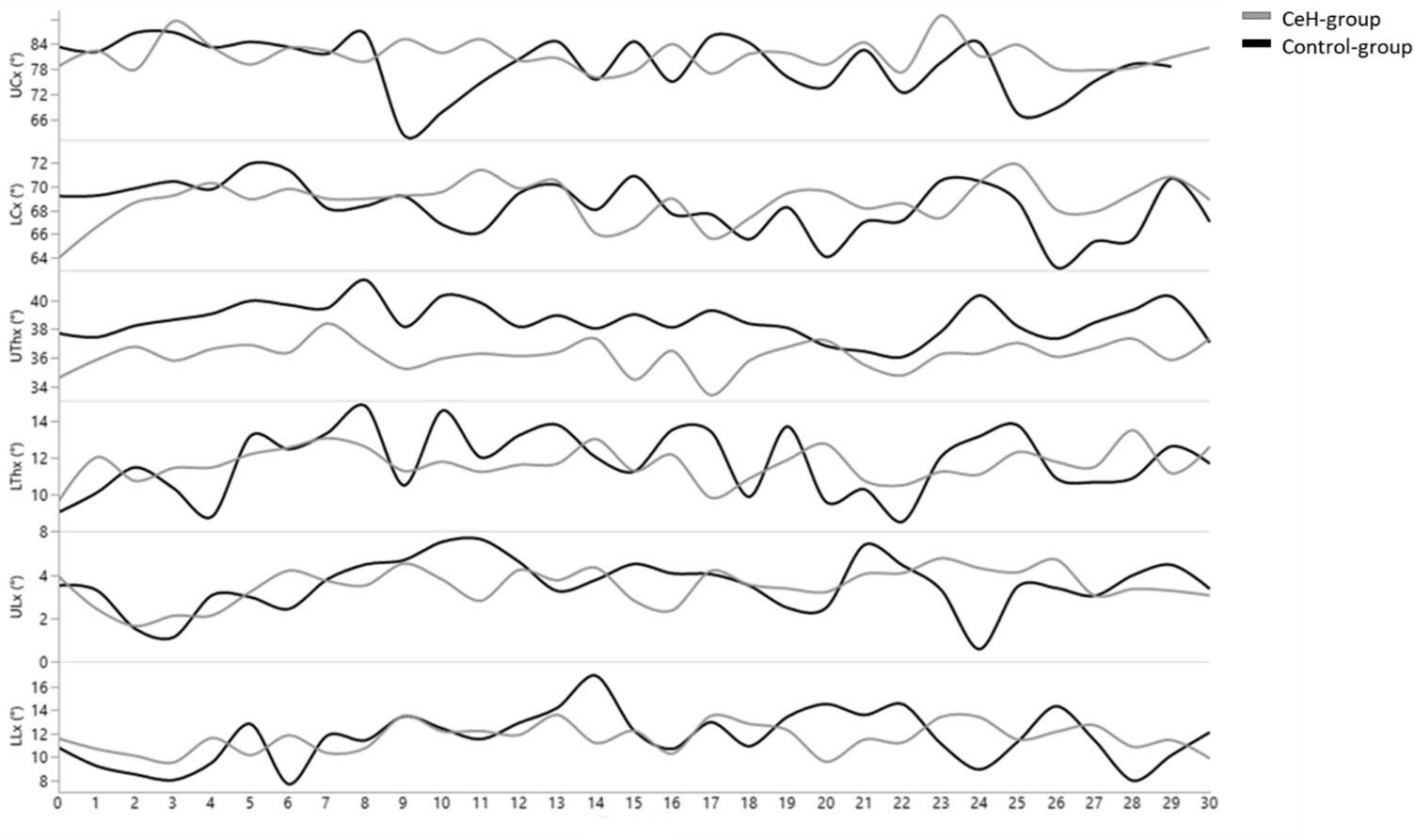
Visualisation of habitual spinal postures during the 30-min-laptop-task in the CeH-group and the control-group.

### Habitual spinal posture

**Figure 3:**
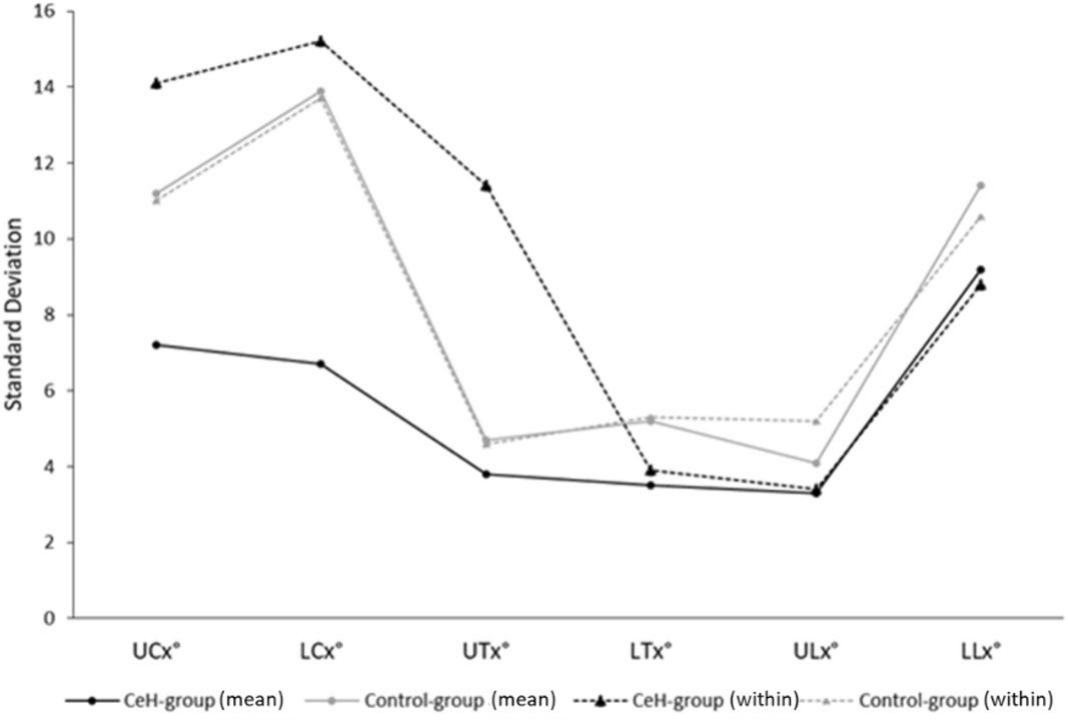
Visualization of the mean and within-group SPV in the CeH-group and control-group expressed through the SD.

Figure 3 visualises habitual spinal postural behaviour of the UCx, LCx, UTx, LTx, ULx, and LLx per minute during the 30-min-laptop-task in the CeH-group (grey) and the control-group (black). The evolution of these habitual spinal postures was rather linear in both groups (time-effect p > 0.05), meaning that time did not influence these postures. Further, none of the six habitual spinal postures differed between the CeH-group and the control-group at t0 and during the 30-min-laptop-task.

### Spinal postural variability (SPV)

The mean SPV differed *between the CeH-group and control-group *at t0 as can be deducted from variability in intercept at t0. This difference was consistent during the 30-min-laptop-task.

*Within the CeH-group,* SPV (quantified by residual variability during 30 measurements) was higher compared to the group’s mean SPV, except for LLx. Concerning upper- vs. lower-spinal variability, UCx- and ULx-variability were lower compared to LCx- and LLx-variability, respectively, and UTx-variability was higher compared to LTx-variability.

ICCs ranged between 0.24 and 0.77, indicating poor to good resemblance of individual SPV in the CeH-group.

*Within the control-group,* SPV was more comparable to the group’s mean SPV. Concerning upper- vs. lower-spinal variability, UCx-, UTx-, and ULx-variability were lower compared to LCx-, LTx-, and LLx-variability respectively.

ICCs ranged between 0.81 and 0.99, indicating good to excellent resemblance of individual SPV in the control-group.

### Does headache-intensity increase after the laptop-task?

Mean headache-intensity (SD) was significantly (p < 0.0001, ES 0.89) higher post laptop-task [NPRS 3.6 (2.1)] compared to pre [NPRS 0.7 (1)] in the CeH-group (Table 2). This difference is larger than the minimal clinical meaningful difference of 2.5^31^.

Pain did not significantly influence UCx (p 0.63), LCx (p 0.55), UTx (p 0.87), LTx (p 0.89), ULx (p 0.61), and LLx (p = 0.85) SPV (Appendix c and d).

## Discussion

Selection of the most adaptive postural adjustment to a task or perturbation characterizes adaptive variability^4, 5, 55^. However, if such variability fails, MSDs, as observed in episodic CeH, can develop^1, 2, 17, 18, 56^. Therefore, the objective of our study was to evaluate SPV in patients with episodic CeH compared to controls.

### Looking beyond static cervical spinal posture in patients with cervicogenic headache: spinal postural variability

In this study, mean habitual spinal postures (= the position of the spine) did not differ between the CeH-group and the control-group, nor did such postures change during the 30-min-laptop-task within each group.

Spinal postures are often used as measurement variable to define the ideal or neutral sitting posture, in which spinal structures are minimally stressed^57^. However, to date, there still is no consensus on the definition of the ideal sitting posture^35, 58^ and attention is shifting toward analysing SPV in the specific context of MSDs^56, 59, 60^.

SPV, i.e. variation in the mean habitual spinal posture which clinically implies postural adjustments in the motor constituents of a task, is important for adaptation to the task demands^1^. Constrained postures with insufficient variation are risk factors to generate MSDs^17, 18^. Therefore, increasing postural variation to decrease biomechanical workload is currently the most suggested preventive intervention for the development of such MSDs^61^.

Several measurement variables can be used to express SPV. Counting the frequency of postural change approaches the time-domain, sampling entropy the complexity of time-series data, evaluating ranges or variance (and the derived SD) the magnitude of SPV^61–63^. We used the SD as main measure to estimate SPV since mean spinal postures were not subject to a time-effect.

Besides determining the most appropriate measurement variable we encountered a second methodological issue when analysing SPV. Comparing the mean SPV between-groups might be misleading since it could mask individual differences. Several authors stress the need to address differences between individuals, and not between groups, since individuals performing an identical task, use different motor patterns^2^.

Thus, although it seems relevant to analyse SPV in case of MSDs, such variability needs to be explored further.

### Spinal postural variability elucidated

What would have been concluded based on this study if we only had analysed differences in the mean SPV between the CeH-group and control-group?

In our study, the mean SPV of the upper- and lower-cervical, upper- and lower-thoracic, and upper- and lower-lumbar spine was smaller in the CeH-group compared to the control-group, both at t0 and during the laptop-task. Such postural behaviour might be a protective strategy, consistent with the contemporary theory of motor adaptations to pain^22^. The latter suggests that pain has a general aim to protect the painful or threatened part of the body from real or impending pain by constraining movement. Such adaptations may even occur in regions remote to the site of pain^22^ as was shown by Falla et al. (2017)^64^. In that study participants with neck pain walked with a stiffer (i.e. decreased rotation) trunk. Applied to our results, this theory might imply that patients with episodic CeH want to protect their cervical spine by additionally stiffening the adjacent thoracic and lumbar region. Although decreasing SPV might be successful at short-term, at long-term (i.e. being unable to recapture variability) such behaviour is contra-productive. A minimum degree of SPV creates variation in joint load, muscle activity, and ligament stress as was postulated by the ‘variability-overuse hypothesis’^22–24, 65^.

Based on the lower cervical, thoracic and lumbar mean SPV in the CeH-group during the laptop-task, a cumulative load on the upper-cervical spine might activate the final common pathway (i.e. trigeminocervical nucleus caudatus) to CeH^66^.

Although our findings might give the impression that future research should build on a general decreased SPV in episodic CeH, by averaging and only reporting data on group level important postural information might be missed. It is for instance not possible to differentiate between extremes in SPV or detect specific patterns within a group^67^. In general, it is presumed that increased postural variability is a healthier state while decreased postural variability is related to MSDs. It should however be kept in mind that ‘too much’ postural variability can also result in overuse-injuries^56^. Finding a balanced SPV might reflect an individual’s self-regulatory capacity to function in an optimal individual range of postural variability. Within such optimum the window of adaptive postural variability should be determined to prevent overuse-injuries from developing^56^.

This hypothesis is deducted from the process of homeostasis: *‘the processes whereby the internal environment of an organism tends to remain balanced and stable’*^68^, critical to the sustainability of living organisms^68^. Through feedback loops, systems are regulated and controlled to maintain the best state upon the influence of various disturbances. Variability is the key factor in sustainability, too much or little, or hyper- versus hypo-variability, could however be harmful^69^.

Therefore, we propose to further explore SPV within the CeH-group. Based on our results, we cannot assume that each participant with episodic CeH used an identical motor strategy, which was— at group level – a decreased SPV. Results in the CeH-group concerning within-group-variability (i.e. difference in variability caused by differences within one group) and ICC point in the direction of more heterogeneity within the CeH-group compared to the control-group. Such findings are consistent with previous work in which it was stated that motor behaviour, as a response or anticipation to pain, is not always stereotypical or even predictable^2^.

Changes in motor behaviour are unique, and postural responses to pain can be highly variable between and probably within individuals^22, 70, 71^. More research is needed to determine if an individual range of optimal SPV exists, and if moving outside such range increases the risk of developing MSDs. This hypothesis supports the importance of an individual approach which already was accentuated by results of Srinivasan and Mathiassen (2012)^2^. From these findings it may be questioned whether individual differences in SPV could predict if someone is more susceptible to develop MSDs^72^. If SPV is a protective factor against the development of MSDs^2^, factors that determine such variability should be explored.

### Upper vs. lower spinal postural variability

It seems relevant to further explore if differences between upper and lower SPV might contribute to or protect against CeH.

Differences in upper- and lower-cervical postural variability have been suggested as a strategy to minimize load on cervical structures^73–75^. Anatomical and physiological evidence suggest greater contribution to sensorimotor control of cervical afferents from the upper- compared to lower-cervical spine^76, 77^. Since sensorimotor control and pain are bi-directionally related, pain can be both cause or be the consequence of disturbed sensorimotor control. This disturbance has an adverse influence on feedback and feedforward motor control and regulation of muscle stiffness^70^.

Additionally, such differences between upper and lower SPV might be extended to the thoracic and lumbar spine since spinal movement during functional tasks is not confined to exclusive the cervical, thoracic or lumbar spine^21^. High inter-regional movement coordination between the cervical and upper-thoracic spine for instance supports such idea^75^. In our study, lower-cervical postural variability was higher compared to upper-cervical postural variability, and upper-thoracic postural variability higher compared to lower-thoracic postural variability in the CeH-group. However, more research is needed to determine if such finding can be related to development of headache. Analysing if SPV could be an outcome measure for a longitudinal interventional study seems a next future research step.

### Headache-intensity versus spinal postural variability

An obvious association to explore would be the relation between pain and decreased SPV. Several authors support the hypothesis that decreased motor variability is the motor response of patients suffering from long-term pain conditions (e.g. low back pain, unilateral patella-femoral pain, spastic hemiplegic cerebral palsy) trying to limit their movement as a protective mechanism^78–81^. We, however, cannot confirm that headache was the main driver for our results since a condition to be measured was a score of < 3 on the 11-point NPRS.  It should also be kept in mind that participants with CeH suffer from an episodic disorder, in contrast to long-term pain conditions in previous work^78–81^, and headache-intensity was only questioned at the start and end of the laptop-task, not during the task. Headache-intensity increased significantly (p < 0.0001, ES 0.89) after the laptop-task in the CeH-group, scores on the 11-point NPRS  evolved from 0.7 (1) before to 3.6 (2.1) at the end. This difference is larger than the minimal clinical meaningful difference of 2.5^31^.

While all individuals perform an identical activity with some degree of motor variability, the extent of such variability differs between individuals^25, 72, 82^. Such differences, even between individuals of a homogeneous (age, gender, body composition) group, can for instance be due to individual anatomical and physiological characteristics^25^. Further, also psychological characteristics seem to influence motor variability^25^.

In summary, if motor variability is a personal trait linked to episodic CeH, determinants of such variability should be further explored.

### Limitations and suggestions

The current work is a first but essential step to explore SPV in patients with episodic CeH. No Bonferroni corrections were applied because of the explorative nature of our study. Therefore, and because of spectrum bias, results should be interpreted with caution. The small sample size (n=36) in this study is a limitation. Future research should consider larger cohorts.. 

SPV can be assessed at different levels. We used higher level aspects of movement performance (i.e. kinematic models). It seems advised to additionally add lower level aspects (i.e. muscle activation)^25^.

More outcomes, relevant in analysing motor variability, need to be explored (e.g. fatigue, anatomical, physiological and psychosocial individual characteristics).

Longitudinal research is further needed to analyse factors that might be associated with SPV within a biopsychosocial framework, and the direction of causality before this knowledge can be integrated into guidelines for evidence-based clinical practice for CeH. In a step wise future process, case–control (n = 1) studies^83^ could be the next step to accentuate the individual aspect of SPV Performing an identical measurement during a headache-attack would be an interesting future step which needs to be well designed. Standardizing an identical level of headacheintensity, because of variability, will be a challenge^84^.

## Conclusion

The mean SPV was lower in the CeH-group compared to the control-group at t0 and during the laptop-task. However, the higher within-group-variability in the CeH-group as opposed to the group’s mean SPV accentuated the heterogeneous postural profile, and might indicate that SPV is a personal trait.

## Supplementary Information

Supplementary Information

## Abbreviations

BMI: Body mass index
CeH: Cervicogenic headache
FHP: Forward head posture
ICC: Intraclass correlation coefficient
ICHD: International Classification of Headache Disorders
LCx: Lower-cervical spine
LLx: Lower-lumbar spine
LTx: Lower-thoracic spine
MSD: Musculoskeletal disorder
NPRS: Numeric Pain Rating Scale
SD: Standard deviation
SPV: Spinal postural variability
TCC: Trigemino-cervical complex
UCX: Upper-cervical spine
ULx: Upper-lumbar spine
UTX: Upper-thoracic spine
