# Supplementary material for: Lower spinal postural variability during laptop-work in subjects with cervicogenic headache compared to healthy controls
Source: Sci Rep. 2021 Mar 4;11:5159. doi: 10.1038/s41598-021-84457-6 (PMC7933416; doi:10.1038/s41598-021-84457-6)
Supplement: Supplementary file 1 — Supplementary Information [file 41598_2021_84457_MOESM1_ESM.docx]

**Lower spinal postural variability during laptop-work in subjects with cervicogenic headache compared to healthy controls**

Sarah Mingels^1,2*^, Wim Dankaerts^2^, Ludo van Etten^3^, Liesbeth Bruckers^4^, and Marita Granitzer^1^

^1^ REVAL Rehabilitation Research Centre, Biomedical Research Institute, Faculty of Rehabilitation Sciences, Hasselt University, 3500, Hasselt, Belgium

^2^ Musculoskeletal Research Unit, Department of Rehabilitation Sciences, Faculty of Kinesiology and Rehabilitation Sciences, Leuven University, 3000, Leuven, Belgium

^3^ Department of Biometrics, Zuyd Hogeschool, 6419, Heerlen, The Netherlands

^4^ Interuniversity Institute for Biostatistics and Statistical Bioinformatics, Hasselt University, 3500, Hasselt, Belgium

* corresponding author: [sarah.mingels@uhasselt.be](mailto:sarah.mingels@uhasselt.be)

**Appendix a – Flowchart of the recruitment and enrolment procedure**

***
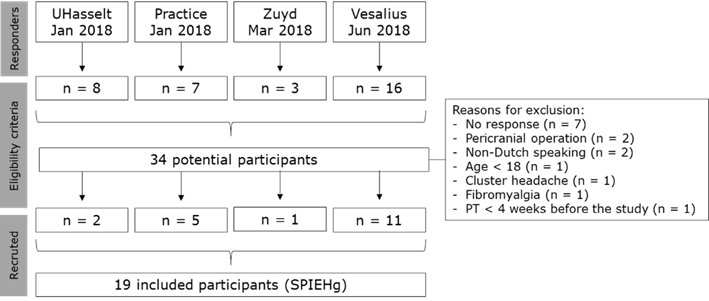
***

CeH-group

**Figure a.1.** Flowchart of recruitment of the CeH-group (n = number responders; PT = Physiotherapy).


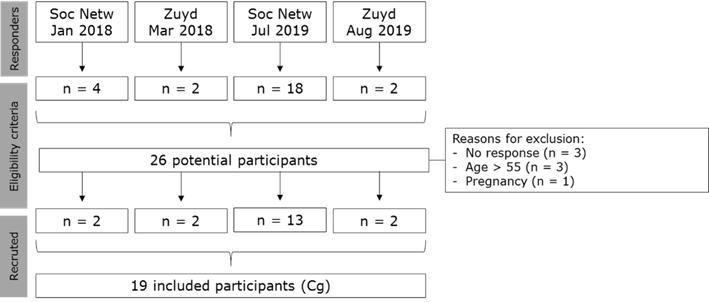


(Control-group)

**Figure a.2.** Flowchart of recruitment of the control-group (n = number responders; Soc Netw = Social Network).

**Appendix b – Details on statistics**

***General form of the mixed model (Eq. b.1)***

Y_ij_ = β_0_ + β_1_X_1ij_ + … + β_k_X_kij_ + μ_i_ + ε_ij_

Y_ij_: value of the response variable for the *i*^th^ observation at *j*^th^ time

β_0_: intercept

X_1ij_: fixed effect design matrix for the *i*^th^ observation at *j*^th^ time

β: regression coefficient independent variable

k: number of independent variables

μ_i_: random effect design matrix for the *i*^th^ observation

ε_ij_: vector of error components for the *i*^th^ observation at *j*^th^ time, ε_i_ ~ N(0,Σ)

β_1_, …, β_k_, ε_i_, …, ε_k_ are independent

_i_: patient

_j_: patient

***Effect of independent variables (Eq. b.2)***

Postural angle_i_ = β_0_ + β_1_age_i_ + β_2_BMI_i_ + β_3_(age x BMI)_i_ + β_4_level of education_i_ + β_5_employment_i_ + ε_i_. The general form of the multiple regression model is given as:

Y_i_ = β_0_ + β_1_X_i1_ + β_2_X_i2_ + β_3_X_i1_X_i2_ + … + β_k_X_ik_ + ε_i_

Y_i_: value of the response variable for the *i*^th^ observation

β_0:_ intercept

X_i_: the matrix of covariates

X_i1_X_i2_: interaction term

β_1_: regression coefficient explanatory variable for *i*^th^ observation, β_i_ ~ N(0,D)

k: number of independent variables

ε_i_ : vector of error components, ε_i_ ~ N(0,Σ)

β_1_, …, β_k_, ε_i_, …, ε_k_ are independent

_i_: patient

_j_: time

**Appendix c – Relations between the independent variables and the outcomes**

The relations between of age, BMI, their interaction (age x BMI), level of education and employment and spinal posture angles were analyzed through multiple linear regression (continuous outcome). An overview of the statistical significance (p-values) of these relations is provided in Tables c.1 and c.2.

**Table c.1.** Summary of relations between the independent variables and the outcomes in the CeH-group.

| Outcome | Age (p) | BMI (p) | Age x BMI (p) | LOE (p) | Job (p) | Headache-intensity (p) |
| --- | --- | --- | --- | --- | --- | --- |
| SPINAL SITTING POSTURE CHARACTERISTICS | | | | | | |
| Hab UCx | .13 | .07 | .19 | .87 | .94 | - |
| Hab LCx | .75 | .88 | .31 | .9 | .53 | - |
| Hab UTx | .49 | .74 | .27 | .63 | .78 | - |
| Hab LTx | .57 | .72 | .15 | .36 | .32 | - |
| Hab ULx | .9 | .44 | .15 | .53 | .4 | - |
| Hab LLx | .89 | .6 | .18 | .18 | .51 | - |
| SD UCx | .81 | .11 | .79 | .72 | .92 | .63 |
| SD LCx | .46 | .45 | .6 | .09 | .47 | .55 |
| SD UTx | .87 | .76 | .62 | .91 | .71 | .87 |
| SD LTx | .62 | .34 | .82 | .47 | .45 | .89 |
| SD ULx | .65 | .9 | .83 | .77 | .66 | .61 |
| SD LLx | .67 | .34 | .17 | .77 | .44 | .85 |

Hab = Habitual posture at t0; p-values were deducted from multiple regression models; SD = Standard Deviation; LOE = Level of Education; - = not the scope of the study; Headache-intensity = NPRS post-laptop-task.

**Table c.2.** Summary of relations between the independent variables and the outcomes in the control-group.

| Outcome | Age (p) | BMI (p) | Age x BMI (p) | LOE (p) | Job (p) |
| --- | --- | --- | --- | --- | --- |
| SPINAL SITTING POSTURE CHARACTERISTICS | | | | | |
| Hab UCx | .93 | .7 | .41 | .11 | .46 |
| Hab LCx | .91 | .64 | .74 | .91 | .77 |
| Hab UTx | .49 | .9 | .79 | .61 | .11 |
| Hab LTx | .53 | .1 | .7 | .33 | .31 |
| Hab ULx | .8 | .26 | .94 | **.001^‡^** | .76 |
| Hab LLx | .1 | .44 | .58 | .2 | .09 |
| SD UCx | .62 | .22 | .05 | .62 | .16 |
| SD LCx | .11 | .72 | .05 | .33 | .08 |
| SD UTx | .85 | .85 | .83 | .99 | .99 |
| SD LTx | .05 | .06 | .36 | .69 | .05 |
| SD ULx | .05 | .55 | .55 | .95 | .1 |
| SD LLx | .46 | .28 | .05 | .21 | .62 |

Hab = Habitual posture at t0; p-values were deducted from multiple regression models; Bold numbers = p < .05; SD = Standard Deviation; ^‡^ = level of education (high school, n = 2) was significantly related (estimate 16) to more ULx flexion; LOE = Level of Education.

**Appendix d – Details mixed models**

**Table d.1.** Summary of the outcomes of the mixed model for repeated measures.

| UCx CeH-group Control-group p ^Δ^ | | | |
| --- | --- | --- | --- |
| Baseline habitual UCx (°), (SD) [CI]  Covariance parameters, estimate (SD) [CI]  Intercept  Time  Variance  Structure (AR)1 | 77.9 (15.6) [69.6;86.2]  10.83 (8.43) [3.61;124.91]  0.05 (0.03) [0.02;0.26]  51.93 (4.16) [44.65;61.16]  0.33 (0.05) [0.24;0.43] | 82.4 (14.3) [75.;89.8]  73.01 (35.58) [33.85;259.69]  0.08 (0.05) [0.03;0.49]  125.58 (8.44) [110.56:143.91]  0.12 (0.05) [0.02;0.21] | .4^‡^ |
| Fixed effects, estimate (SD)  Intercept  Baseline  Time  Group  Group x time (Control-group) | 29.46 (10.23)  0.67 (0.12)  -0.08 (0.08)  -1.12 (2.7)  0.01 (0.12) | | .**008**  **< .0001**  .3  .68  .91 |
| LCx CeH-group Control-group p ^Δ^ | | | |
| Baseline habitual LCx (°), (SD) [CI]  Covariance parameters, estimate (SD) [CI]  Intercept  Time  Variance  Structure (AR)1 | 63.9 (16) [56;71.9]  29.84 (14.02) [14.15;98.88]  0.04 (0.03) [0.01;0.26]  45.31 (3.56) [39.07;53.17]  0.32 (0.05) [0.22;0.42] | 66.5 (12.3) [60.1;72.8]  61.16 (31.9) [27.16;242]  0.02 (0.04) [0.001;1.1]  193.11 (13.25) [169.55;221.97]  0.24 (0.04) [0.15;0.33] | .6^†^ |
| Fixed effects, estimate (SD)  Intercept  Baseline  Time  Group  Group x time (Control-group) | 32.24 (55.96)  0.55 (0.09)  0.07 (0.07)  -2.88 (2.92)  0.56 (0.09) | | **< .0001**  **< .0001**  .27  .33  .69 |
| UTx CeH-group Control-group p ^Δ^ | | | |
| Baseline habitual UTx (°), (SD) [CI]  Covariance parameters, estimate (SD) [CI]  Intercept  Time  Variance  Structure (AR)1 | 33.8 (10.7) [28.3;39.3]  11.88 (5.36) [21.66;101.35]  0.03 (0.02) [0.02;0.10]  14.23 (1.05) [12.39;16.53]  0.25 (0.05) [0.15;0.35] | 37.8 (8.7) [33.3;42.2]  40.52 (15.39) [5.76;37.14]  0.04 (0.02) [0.02;0.11]  21.56 (1.46) [18.97;24.75]  0.18 (0.05) [0.08;0.27] | .25^†^ |
| Fixed effects, estimate (SD)  Intercept  Baseline  Time  Group  Group x time (Control-group) | 11.48 (3.27)  0.73 (0.1)  0.03 (0.05)  -0.2 (1.97)  -0.008 (0.08) | | **.003**  **< .0001**  .56  .92  .91 |

| LTx CeH-group Control-group p ^Δ^ | | | |
| --- | --- | --- | --- |
| Baseline habitual LTx (°), (SD) [CI]  Covariance parameters, estimate (SD) [CI]  Intercept  Time  Variance  Structure (AR)1 | 9.6 (5.8) [6.8;12.5]  1.73 (1.67) [0.49;54.42]  0.03 (0.01) [0.01;0.08]  12.16 (1) [10.41;14.38]  -0.04 (0.05) [0.29;0.48] | 8.8 (7.4) [4.9;12.8]  10.06 (4.42) [4.96;30.24]  0.01 (0.007) [0.004;0.07]  27.03 (1.71) [23.96;30.71]  0.39 (0.05) [-0.13;0.06] | .72^†^ |
| Fixed effects, estimate (SD)  Intercept  Baseline  Time  Group  Group x time (Control-group) | 2.76 (0.88)  0.91 (0.08)  0.005 (0.05)  -0.03 (0.06)  0.01 (0.02) | | **.004**  **< .0001**  .91  .57  .51 |
| ULx CeH-group Control-group p ^Δ^ | | | |
| Baseline habitual ULx (°), (SD) [CI]  Covariance parameters, estimate (SD) [CI]  Intercept  Time  Variance  Structure (AR)1 | 4.1 (8.5) [-0.3;8.5]  3.25 (1.92) [1.33;16.62]  0.01 (0.006) [0.004;0.05]  10.89 (0.8) [9.47;12.65]  0.3 (0.04) [0.21;0.4] | 7.6 (12.4) [1;14.2]  4.17 (3) [1.48;36.36]  0.01 (0.008) [14.24;19.56]  16.58 (1.34) [14.24;19.56]  0.39 (0.05) [0.3;0.48] | .56^‡^ |
| Fixed effects, estimate (SD)  Intercept  Baseline  Time  Group  Group x time (Control-group) | 0.15 (0.62)  0.77 (0.06)  0.03 (0.05)  1.18 (0.92)  -0.006 (0.05) | | .81  **< .0001**  .46  .21  .9 |
| LLx CeH-group Control-group p ^Δ^ | | | |
| Baseline habitual LLx (°), (SD) [CI]  Covariance parameters, estimate (SD) [CI]  Intercept  Time  Variance  Structure (AR)1 | 11.7 (11.6) [5.5;17.9]  56.02 (21.43) [29.83;141.27]  0  84 (9.16) [72.42;98.62]  1 | 13.9 (13.3) [6.5;21.2]  150.18 (59.55) [78.47;394.88]  0.24 (0.12) [0.11;0.91]  130.77 (11.44) [110.36;157.44]  0.98 | .62^†^ |
| Fixed effects, estimate (SD)  Intercept  Baseline  Time  Group  Group x time (Control-group) | 17.11 (3.84)  -0.04 (0.01)  0.03 (0.07)  -4.14 (4.99)  -0.07 (0.16) | | **< .0001**  **.0007**  .62  .41  .35 |

^‡^ = Mann-Whitney test; ^†^ = unpaired t-test; ^Δ^ = p-value deducted from the mixed model with random and fixed effects; Bold numbers = p < .05.

**Table d.2.** Summary of the influence of headache intensity (NPRS post-laptop-task) on variance in the CeH-group, deducted from the mixed model.

| Spinal angle | Variance (SD) [CI]  Headache intensity included as independent variable | Variance (SD) [CI]  Headache intensity excluded as independent variable |
| --- | --- | --- |
| UCx | 52.11 (4.21) [44.74;61.46] | 51.93 (4.16) [44.65;61.16] |
| LCx | 45.13 (3.52) [38.95;52.91] | 45.31 (3.56) [39.07;53.17] |
| UTx | 14.26 (1.05) [12.4;16.56] | 14.23 (1.05) [12.39;16.53] |
| LTx | 10.55 (1.86) [7.68;15.41] | 12.16 (1) [10.41;14.38] |
| ULx | 10.81 (0.79) [9.42;12.54] | 10.89 (0.8) [9.47;12.65] |
| LLx | 83.84 (6.77) [72;98.88] | 84 (9.16) [72.42;98.62] |
